# Pain and quality of life in patients undergoing radiotherapy for spinal metastatic disease treatment

**DOI:** 10.1186/1755-7682-6-6

**Published:** 2013-02-18

**Authors:** Edgar S Valesin Filho, Luiz Carlos de Abreu, Guilherme HV Lima, Daniel IG de Cubero, Fabrício H Ueno, Gustavo SL Figueiredo, Vitor E Valenti, Carlos Bandeira de Mello Monteiro, Rubens Wajnsztejn, Edison N Fujiki, Modesto Rolim Neto, Luciano M Rodrigues

**Affiliations:** 1 Hospital Estadual Mário Covas, Santo André, SP, Brazil; 2 Laboratório de Delineamento de Estudos e Escrita Científica. Departamento de Morfologia e Fisiologia, Faculdade de Medicina do ABC, Av. Príncipe de Gales, 821, 09060-650, Santo André, SP, Brazil; 3 Faculdade de Filosofia e Ciências, Universidade Estadual Paulista, UNESP. Av. Hygino Muzzi Filho, 737, 17.525-900, Marília, SP, Brazil; 4 Escola de Artes, Ciências e Humanidades da Universidade de São Paulo, São Paulo, Brazil

**Keywords:** Radiotherapy, Neoplasm metastasis, Quality of life

## Abstract

**Background:**

Radiotherapy is an important tool in the control of pain in patients with spinal metastatic disease. We aimed to evaluate pain and of quality of life of patients with spinal metastatic disease undergoing radiotherapy with supportive treatment.

**Methods:**

The study enrolled 30 patients. From January 2008 to January 2010, patients selection included those treated with a 20 Gy tumour dose in five fractions. Patients completed the visual analogue scale for pain assessment and the SF-36 questionnaire for quality of life assessment.

**Results:**

The most frequent primary sites were breast, multiple myeloma, prostate and lymphoma. It was found that 14 spinal metastatic disease patients (46.66%) had restricted involvement of three or fewer vertebrae, while 16 patients (53.33%) had cases involving more than three vertebrae. The data from the visual analogue scale evaluation of pain showed that the average initial score was 5.7 points, the value 30 days after the end of radiotherapy was 4.60 points and the average value 6 months after treatment was 4.25 points. Notably, this final value was 25.43% lower than the value from the initial analysis. With regard to the quality of life evaluation, only the values for the functional capability and social aspects categories of the questionnaire showed significant improvement.

**Conclusion:**

Radiotherapy with supportive treatment appears to be an important tool for the treatment of pain in patients with spinal metastatic disease.

## Background

Spinal metastatic disease (SMD) is a relatively common disease and has received increased attention recently with the development of diagnostic and therapeutic tools that increase the life expectancy of these oncology patients [1,2]. Radiotherapy is an important tool for the control of pain and local neoplastic progression. Several studies have demonstrated its efficacy in stabilizing the progression of pain and in maintaining the ability of the patient to walk, which are considered import goals of treatment [3-6].

The majority of tumors found in the spine are metastatic lesions, and approximately 18,000 new cases are diagnosed annually in the United States [7]. According to the literature, more than 10% of patients with cancer will develop symptomatic secondary spinal disease, and more than 40 to 70% of those cases will involve several vertebral levels [8-10].

Historically, radiotherapy has been used in the treatment of SMD and is considered the standard treatment by many authors [11-13]. Several studies have shown only negligible benefits from decompressive surgery (i.e., isolated laminectomy), associated or not with radiotherapy, when compared to radiotherapy alone for the treatment of pain and neurological dysfunction in patients [14]. The current dogma that dictates surgery for spinal metastatic disease as the procedure of choice to save the life of a patient has been established for some time.

However, previous studies have considered laminectomy inefficient in decompressing and resecting a tumor mass that is found in the vertebral body in most cases. Moreover, this procedure can lead to vertebral instability and deformities as well as the possible neurological and clinical deterioration of the patient. With recent advances in surgical techniques and the development of less invasive procedures, as well as earlier and more precise diagnosis, surgical approaches have shown improved outcomes in treating oncology patients, thereby making surgery a first-line treatment in selected cases [15].

Radiotherapy is still a fundamental therapeutic option for neoplasms sensitive to this treatment, such as lymphomas and multiple myelomas. This option is most commonly used with patients who have lesions at multiple levels or where surgery would be contraindicated by the clinical condition of the patient. There is still controversy with regard to the benefits of surgery, radiotherapy, chemotherapy or any combination of these treatments for different groups of patients [16].

Although previous studies have already investigated the radiation therapy effects on metastasis [17,18], its effects on immunological responses are hypothesized to impair the quality of life. Therefore, we aimed to analyze the progression of pain and of quality of life in patients undergoing radiotherapy with supportive treatment to treat SMD-related pain.

## Methods

All patients considered for this study were diagnosed with SMD between January 2008 and January 2010. A total of 30 patients undergoing radiotherapy for pain management and to prevent the local progression of the tumor were included in the study. Patients that had previous decompressive or stabilization surgeries in addition to receiving radiotherapy were excluded.

Clinical outpatient evaluation was performed with the help of the Clinical Oncology team of the same service. Questionnaires were administered by the treating orthopedic physician specialized in spinal surgery. Patients enrolled in the study provided their informed and written consent. All procedures were approved by the Ethics Committee in Research of our University.

Details of other supportive treatments other than surgery and radiation therapy included steroid therapy (dexamethasone, 4 mg every 6 hours), spinal orthotics and physiotherapy.

The criteria used for radiotherapy indication were the following: patients selection included those treated with 20 Gy tumour dose, provided in five fractions, control of local pain that was not treatable by medication, patients with lesions in multiple levels, patients with pain and complete neurological deficits for more than 48 hours after neoplastic spinal cord compression and tumors sensitive to radiotherapy in patients without progressive neurological alteration during treatment. All patients were sent to the same radiotherapy clinic and underwent treatment with fractionated doses according to a specific protocol (20 Gy tumour dose in five fractions).

The questionnaire evaluated the pain in SMD subjects and its impact on quality of life at three different times: before radiotherapy, 30 days after the end of the treatment and 6 months after the end of the treatment. The visual analogue scale (VAS) [19] was used to measure pain, and the SF-36 questionnaire [20,21] was used to study possible quality of life variations. The SF-36 consists of 36 items that are grouped into the following eight areas: functional capacity, physical limitations, pain, overall health, vitality, social aspects, emotional limitations and mental health. Results were statistically evaluated using SPSS software (Statistical Package for Social Sciences, version 17.0) Statistical Package for Social Sciences software (SPSS, version 17.0) and were considered significant for p < 0.05.

## Results

**Figure 1:**
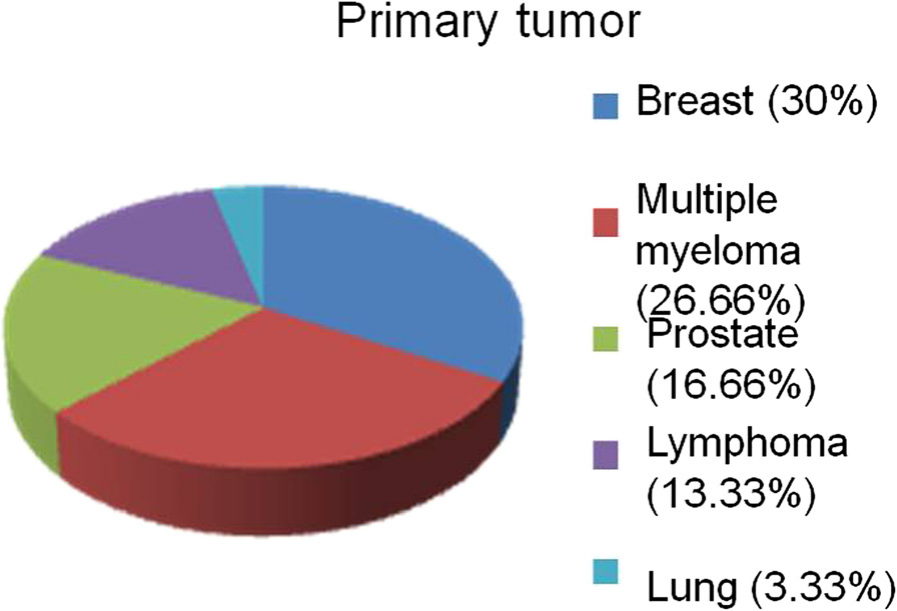
Distribution of the primary sites of metastases.

Thirty patients were chosen for the study, including 18 women (70%) and 12 men (30%). All patients participated in the evaluation performed 30 days after the end of radiotherapy. However, by the 6-month evaluation, there were 10 deaths, thereby resulting in a survival rate of 66.66%. The distribution of the primary sites of neoplasm afflicting these patients is presented in Figure 1. The patients were aged between 40 and 90 years old with an average age of 62.2 years old.

SMD involving three or fewer vertebrae was found in 14 cases (46.66%), whereas SMD that had disseminated (i.e., lesions in more than three vertebrae) was found in 16 patients (53.33%). Topography characteristic of restricted lesions was more frequently found in the thoracic spine (i.e., 8 cases, or 57.14% of the restricted lesions), although in 5 cases (35.71%) the lesions occurred in the lumbar spine. In 1 case (7.14%), the lesion occurred in the cervical spine.

**Figure 2:**
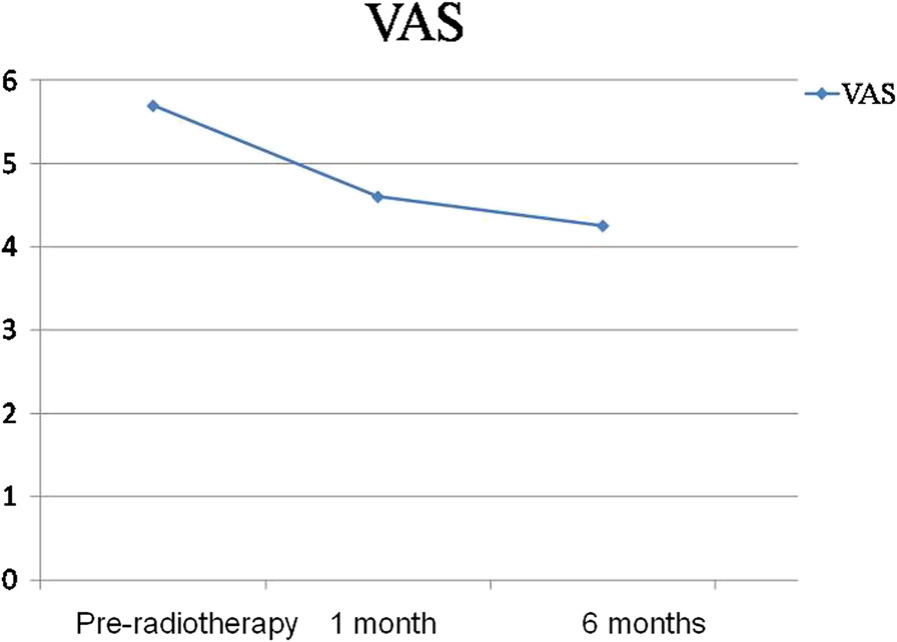
Average pain score according to the VAS.

The evaluation of pain according to the visual analogue scale (VAS) yielded an initial average value of 5.7 points. Thirty days after the end of radiotherapy, this was found to be 4.6 points on average. Six months after treatment, the average VAS score was 4.25 points (Figure 2), which is 25.43% lower than the initial value.

To describe and compare the observational periods regarding the variable VAS, we applied the *Friedman test.* The goal of this analysis was to verify possible differences among the three time points of observation (Table 1). Patients that died during the study were not included in this analysis.

**Table 1:** Values of VAS for the analyzed time points

***Variables***	***n***	***Average***	***Standard deviation***	***Min***	***Max***	***Median***
***VAS pre radiotherapy***	20	5.7	3.05	0.00	10.00	6.5
***VAS 1 month post radiotherapy***	20	4.6*	2.52	0.00	10.00	5.00
***VAS 6 months post radiotherapy***	20	4.25*	3.16	0.00	10.00	4.00

*p=0.002: Different from VAS pre radiotherapy.

Because we found significant difference between the three testing time points, we applied the *Wilcoxon test*, which was adjusted by a *Bonferroni correction*, in order to identify the observational periods that were significantly different (Table 2).

**Table 2:** Differences between the VAS values when comparing the time points analyzed in pairs

***Pair of variables***	***p***
VAS 1 month post radiotherapy vs. VAS pre radiotherapy	0.010
VAS 6 months post radiotherapy vs. VAS pre radiotherapy	0.009
VAS 6 months post radiotherapy vs. VAS 1 month post radiotherapy	0.546

(alpha for Bonferroni = 0.016952).

We observed the main changes in the VAS values when the pre-radiotherapy period was paired with the consecutive follow-up analyses, individually. Nevertheless, no statistically significant alteration in the VAS scores was found when the 30-day post radiotherapy and 6-month post radiotherapy time points were compared.

To complete the quality of life evaluation using the SF-36 questionnaire, we compared the eight distinct subject areas across the observation time points. We applied the *Friedman test* to verify possible differences among the three observation time points.

**Figure 3:**
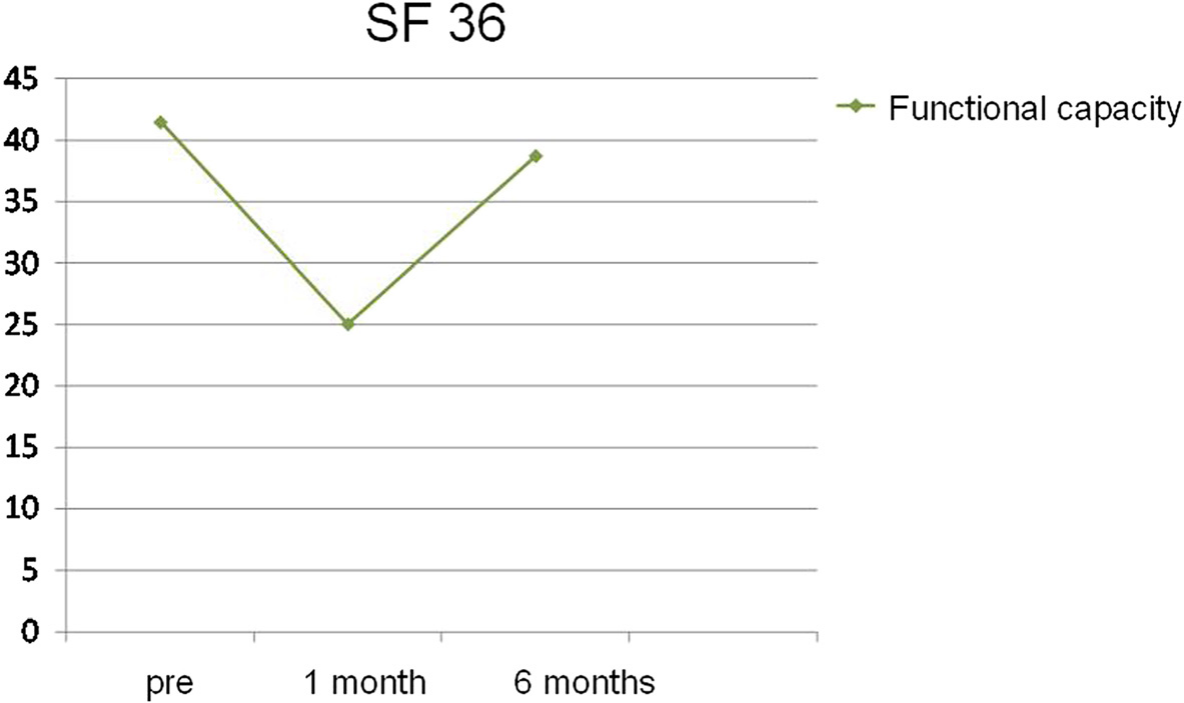
Variation in the area of functional capacity using the SF-36.

**Figure 4:**
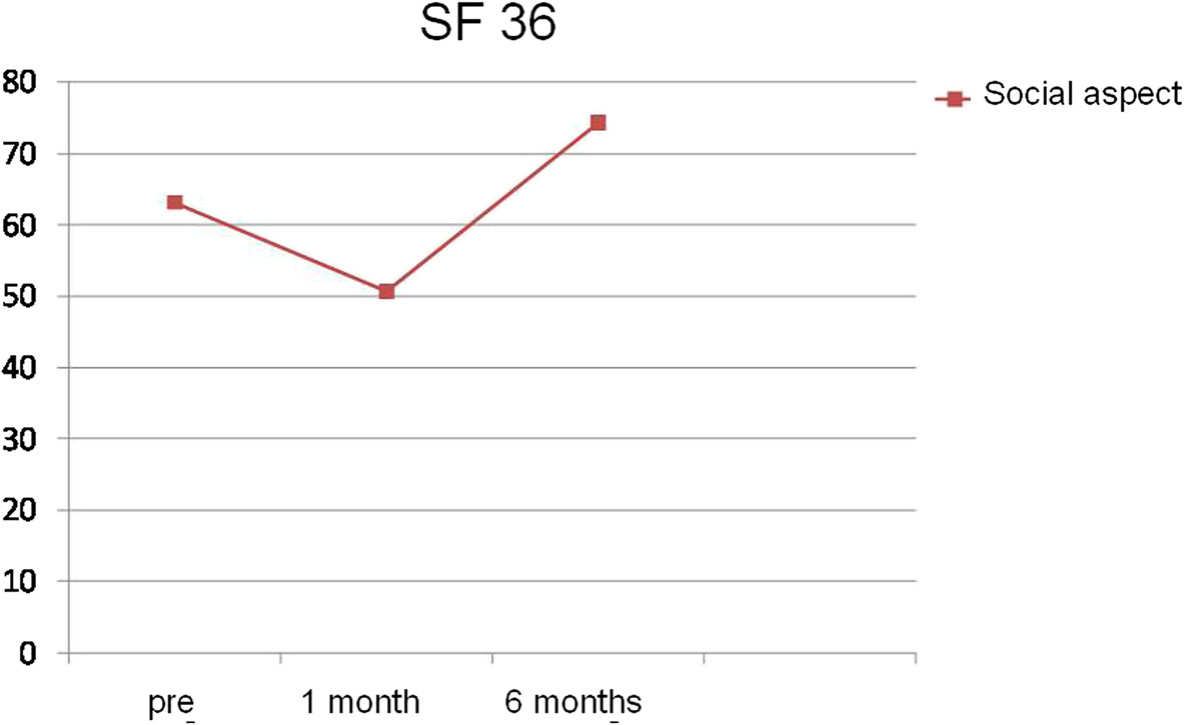
Variation in the area of social aspects using the SF-36.

Only in the areas of functional capacity (Figure 3) and social aspects (Figure 4) we found significant higher values at pre and 6 months post radiotherapy compared to 1 month post radiotherapy.

We found significant differences in these areas and we compared the three periods concomitantly. We applied the *Wilcoxon test*, which was adjusted by a *Bonferroni* correction in order to identify which observation points where significantly different (Tables 3 and 4).

**Table 3:** Wilcoxon test to evaluate the area of functional capacity at different time points

***Pairs of variables***	***p***
SF-36 functional capacity 1 month post radiotherapy vs. SF-36 functional capacity pre radiotherapy	0.067
SF-36 functional capacity 6 months post radiotherapy vs. SF-36 functional capacity pre radiotherapy	0.690
SF-36 functional capacity 6 months post radiotherapy vs. SF-36 functional capacity 1 month post radiotherapy	0.011

(alpha value for Bonferroni = 0.016952).

**Table 4:** Wilcoxon test to evaluate the area of social aspects at different time points

***Pairs of variables***	***p***
SF-36 social aspects 1 month post radiotherapy vs. SF-36 social aspects pre radiotherapy	0.196
SF-36 social aspects 6 months post radiotherapy vs. SF-36 social aspects pre radiotherapy	0.095
SF-36 social aspects 6 months post radiotherapy vs. SF-36 social aspects 1 month post radiotherapy	0.001

(alpha value for Bonferroni = 0.016952).

The average score for functional capacity 6 months after radiotherapy was 38.75 points. This value is significantly higher than the average value obtained for functional capacity 30 days after the end of radiotherapy, which was 25 points. Analogously, the values for social aspects observed at the second and third time points were significantly different. Specifically, the average value for this parameter was 74.38 points 6 months post radiotherapy and was 50.63 points 30 days after the end of treatment.

We also performed statistical analyses to identify possible correlations between changes in pain levels and the SMD being restricted or disseminated, but we did not observe significant differences in the evolution of pain levels when comparing these two groups by either the VAS or SF-36 questionnaires.

## Discussion

In this study we conducted a prospective analysis of pain and quality of life evolution in subjects undergoing radiotherapy with supportive treatment to treat pain caused by spinal metastatic disease. We found that radiotherapy and the related adjuvant treatments significantly improved the progression of pain at 30 days and 6 months after its application in the treatment of SMD.

### Indication

The literature shows that a definitive cure is not an expected outcome for patients with SMD. The main goal of treatment is palliative, and life expectancy is generally short, typically ranging from 4 to 15 months [20,22]. The main symptom of SMD is pain. It is presented as progressive night pains that are generally localized in segments of the thoracic spine with concomitant clinical deterioration. The pathophysiologic mechanisms of osseous pain secondary to neoplasms are multiple and become even more complex in the spine. These underlying factors include chemical mediators, increases in intraosseous pressure, microfractures, periosteum enlargement, pathological fractures and deformities [16,22].

Most patients with SMD and spinal cord compression syndrome (90%) present pain, while 47% present with symptomatic neurological alterations [23]. Moreover, only 11% to 34% of patients with spinal cord compression syndrome are able to walk at the time of diagnosis [24].

Our investigation indicates radiotherapy with supportive treatment as an efficient therapy. According to the literature, radiotherapy is indicated as the first-line therapy for SMD patents in the following situations: when complete neurological deficits are sustained for a period longer than 24 hours, when highly radiosensitive tumors (lymphoma, myeloma) are diagnosed, when there is involvement of multiple levels, when there is short life expectancy (less than 3 months) or when serious co-morbidities contraindicate surgery [15]. Surgery to treat SMD is indicated in specific cases, such as neurologically progressive deficits, pathologic deformities, resistance to radiotherapy and patients with an oncologic prognosis that is favorable.

### Etiology and topography

Currently, there are three theories regarding the origin of extradural metastatic vertebral lesions: (1) progression of local disease, (2) retrograde dissemination via the spinal avalvular venous plexus of Batson and (3) arterial embolisms [25-28].

All the patients’ lesions were evaluated by magnetic resonance in our study and were all demonstrated to be extradural tumors. Classic studies involving autopsy have shown that the distribution of extradural metastases is more common in larger vertebrae. Therefore, the presence of SMD has been documented in the lumbar, thoracic and cervical regions with decreasing frequency [27,29]. Nevertheless, Papadopoulos et al. [28] showed that symptomatic lesions are most frequent in the thoracic region (70%), whereas the lumbar region represents approximately 20% cases and the cervical region about 10% [30]. Notably, our results are in agreement with the literature, whereby patients that presented restricted lesions (i.e., up to three vertebrae involved) were most frequently affected in the thoracic region. Overall, this group comprised 8 cases, or 57.14% of patients. We hypothesize that the small diameter of the vertebral canal in relation to the thoracic spinal cord caused this increase in symptoms [8].

The majority of published studies demonstrated that the major primary sites for extradural vertebral metastasis are the breast, lung and prostate, as well as lymphomas, multiple myelomas and renal cell tumors [27,29-32]. In our study, we observed a predominance of breast lesions (30%), followed by lymphomas and multiple myelomas (40%). Byrne et al. [31] observed that approximately 50% of vertebral metastases originated in one of the three following primary sites: breast, lung or prostate [33]. In a recent review, Klimo et al. [32] suggested that neoplasms originate from the following sites in order of decreasing frequency: renal tumors, gastrointestinal tissue, thyroid tissue, sarcomas and malignancies of lymphoreticular tissue (lymphomas and multiple myelomas) [34].

It is important to mention that the distribution of primary sites related to SMD may vary from one institution to another due to regional epidemiological differences and the specialty of the services involved. Our institution is a regional clinic for hematology, which justifies the greater portion of lymphoma and myeloma cases we encountered when compared to studies from other institutions.

### Evaluation of SMD pain and quality of life with radiotherapy with supportive treatment

The literature suggests that the response to radiotherapy should be characterized in terms of pain relief and functional status [12,13]. In lesions sensitive only to radiotherapy, positive results occur in more than 80% of patients [23,24]. Lower rates of response are observed with lesions that are less sensitive to irradiation. As reported by Jacobs and Perrin [19], more than 30% of patients showed improvement in neurological function due to epidural decompression and more than 60% achieved pain relief after radiotherapy [19,24]. Zaikova et al. [33] conducted a study involving an analysis of pain response among 355 patients that underwent radiotherapy for SMD. After 2 months of treatment, a favorable response (partial or total pain relief) was noted in 37% of patients, which is a lower percentage than is observed with osseous metastasis patients in general. Taken together, these results suggest that the presence of pathophysiological pain is more complex in nature than the pain associated with vertebral metastatic disease.

Chow et al. [34] reported strong positive response rates to radiotherapy in the treatment of osseous metastasis, which ranged from 60 to 90%. Nonetheless, the lowest rates of successful pain relief were observed in patients with vertebral lesions in other skeletal topographies [35,36]. Maranzano et al. administered radiotherapy to treat SMD in 209 patients. After an average of 49 months, 71% of the patients with pain (98% of the studies participants) showed relief from this symptom [13,21,35].

Our investigation recorded the regression of pain with an outpatient evaluation that was quantified by the VAS questionnaire. At 6 months after the radiotherapy, the average score was 25.43% lower than the initial value. This level of pain control is lower than the responses found in the literature. It is possible that the reduced pain control observed in our study was due to the fact that a higher portion of the patients we treated were afflicted with the advanced stages of the disease. The fact that 33% patients died within the 6 months following treatment illustrates this point.

No significant variations in paired evaluations of the data from the 30-day and 6-month post radiotherapy time points were found. On the other hand, there was a significant improvement in pain relief during the 30 days following radiotherapy that evolved over the course of the study.

The analysis of quality of life through the SF-36 questionnaire did not show significant results at the 6-month evaluation period. The only exceptions were in the areas of functional capacity and social aspects, which showed slight improvements when the 6-month time point was compared with the 30-day time point.

Analysis of the pain section of the SF-36 questionnaire did not indicate a significant improvement in pain levels, as was reported by the VAS questionnaire. This difference may have been because the latter consisted of an evaluation guided by the symptoms related to the spine region that was irradiated, while the evaluation of pain according to the SF-36 questionnaire was influenced by symptoms related to the overall clinical deterioration of the patient.

Analgesic medication was also used as an important resource to help control pain. Indeed, several studies have avoided the topic of analgesic usage for the further relief of pain in studies of radiotherapy due to the complexity of the prospective analysis required [2,36,37]. Nevertheless, we did not remove medication from the patients to avoid health’s impairment of the patient.

The standard dose for efficacious irradiation in SMD cases consists of 20 to 30 Gy given over 5 to 10 sessions at the area of vertebral involvement. However, several variations of this regimen have been utilized [38,39]. Although we administered the radiotherapy in fractional doses (20 Gy in five fraction), the majority of studies in the literature do not show differences in pain relief from vertebral metastatic disease when radiotherapy is performed as a single dose or if it is fractionated [37,39,40].

We did not eport significant toxicity in patients that underwent radiotherapy in the present study. Complications from the irradiation of the spine are generally mild. Esophagitis induced by radiation may occur in lesions of the superior thoracic region, while irradiation of the inferior thoracic region and lumbar superior can cause nausea and vomiting. These adverse reactions are more common at the beginning of treatment and are regularly controlled by medication. In contrast, radiation-induced myelopathy is a complication that occurs more commonly in the latter during treatment, and although it is rare, it is extremely serious [37,39,40].

Some points are important to be raised. The supportive treatment other than surgery and radiation therapy included spinal orthotics, dexamethasone and physiotherapy. Unfortunately, we were not able to exclude these variables, since the withdrawal of those treatments would negatively affect the patient’s health. Therefore, we indicate radiation therapy accompanied with those supportive treatment as effective. Although there are specific questionnaire for cancer’s patient, we applied only the SF-36 questionnaire. However, this questionnaire is well accepted in the literature and it was applied in this disease [41-43]. Patients selection included those treated with a 20 Gy tumour dose, provided in five fractions according to a previous study [44]. This method was decided in order to avoid a possible influence of different radiotherapy regimens, i.e. 20 Gy in 5 fractions and 30 Gy in 10 fractions.

Although the use of radiation therapy for bony metastases with or without surgery was presented in previous studies, some studies still considers it as malefic regarding the immunological responses [45,46]. Therefore, our study provided important information to support this type of treatment. Our findings are relevant to the literature, since quality of life is a very important issue to be discussed in order to improve clinical practice [47-51].

## Conclusion

Radiotherapy with supportive treatment resulted in a significant improvement in the progression of pain levels at 30 days and 6 months after its application in the treatment of SMD. The quality of life evaluation showed only significant improvement in functional capacity and social aspects. Thus, radiotherapy with supportive treatment is indicated a useful tool for the control of pain in patients with SMD.

## Abbreviations

SMD: Spinal metastatic disease; VAS: Visual analogue scale

## Competing interest

The authors declare that they have no competing interests.

## Authors’ contribution

ESVF, LCA, GHV, DIGC, FHU, GSLF, VEV, CBMM, RW, ENF, MRN, LMR participated in the acquisition of data and revision of the manuscript. ESVF, GHVL, DIGC, FHU, GSLF and LMRR conceived the study, determined the design, performed the statistical analysis, interpreted the data and drafted the manuscript, VEV, ENF and LCA determined the design, interpreted the data and drafted the manuscript. All authors read and gave final approval for the version submitted for publication.
